# Evaluating COVID-19 Vaccine Willingness and Hesitancy among Parents of Children Aged 5–11 Years with Chronic Conditions in Italy

**DOI:** 10.3390/vaccines10030396

**Published:** 2022-03-04

**Authors:** Grazia Miraglia del Giudice, Annalisa Napoli, Francesco Corea, Lucio Folcarelli, Italo Francesco Angelillo

**Affiliations:** 1 Department of Experimental Medicine, University of Campania “Luigi Vanvitelli”, Via Luciano Armanni 5, 80138 Naples, Italy; grazia.miragliadelgiudice@studenti.unicampania.it (G.M.d.G.); annalisa.napoli@studenti.unicampania.it (A.N.); francesco.corea@studenti.unicampania.it (F.C.); lucio.folcarelli@studenti.unicampania.it (L.F.); 2 Department of Public Health and Laboratory Services, Teaching Hospital of the University of Campania “Luigi Vanvitelli”, Via Luciano Armanni 5, 80138 Naples, Italy

**Keywords:** children, chronic condition, COVID-19, hesitancy, Italy, parents, vaccination

## Abstract

COVID-19 vaccination has been extended to include children aged 5–11 years. This cross-sectional survey evaluated parental COVID-19 vaccine willingness and hesitancy, and associated factors, for their children aged 5–11 years with chronic conditions. A telephone survey was conducted from 14 December 2021 to 4 January 2022. The questionnaire assessed participants’ socio-demographic and health-related information, attitudes towards COVID-19 infection, hesitancy, by using the PACV-5 (Parent Attitudes About Childhood Vaccines Survey Tool), and sources of information. A total of 430 answers were collected anonymously. Respondents with no cohabitant who had been infected by SARS-CoV-2 and having been vaccinated against COVID-19 had a higher concern about the severity of COVID-19. The parents’ perceived risk that the child could be infected by SARS-CoV-2 was higher in those more concerned about the severity of COVID-19, with an older child, and who had at least one cohabitant positive for COVID-19. Only 38.8% parents were willing to vaccinate their children against COVID-19. Parents who did not need additional information, those with higher education, those who have been vaccinated against COVID-19, those whose child was older, who had received information on this vaccination from physicians, with higher self-reported concern about the severity of COVID-19, and who had a higher perceived risk that their child could be infected by SARS-CoV-2, expressed a greater willingness to vaccinate their child. Overall, 26.3% were high-hesitant, with a PACV-5 score ≥ 7. Respondents who did not get the COVID-19 vaccine, were less educated, with a lower concern about severity of COVID-19, and with a lower perceived risk that their child could be infected by SARS-CoV-2, were more likely to be high-hesitant. New policies and educational programs regarding COVID-19 vaccination for children with chronic conditions are needed to reduce hesitancy and increase vaccination uptake.

## 1. Introduction

Since the announcement of the emergence in Wuhan, China, of the novel coronavirus disease 2019 (COVID-19) in December 2019, the disease has continued to spread and cross international borders, with over 308 million cases and 5.4 million deaths, with 22 million (17%) cases reported in children [1,2]. Although COVID-19 is generally milder in children and adolescents than in adults, severe disease and long-term complications can occur after the primary infection and comorbidity conditions are a risk factor [3,4].

Most Western governments have issued effective public health instructions on how to prevent and reduce the diffusion of SARS-CoV-2, initially mainly through non-pharmaceutical recommendations and, from December 2020, with a safe and effective vaccination. In Italy, the COVID-19 vaccination strategy was to propose the vaccine to everyone progressively, according to the individual’s level of risk. From 16 December 2021, as part of the national immunization program, vaccination has been extended to include children aged 5–11 years, and the protocol recommends two doses of the BNT162b2 vaccine, administered 21 days apart [5].

Willingness and hesitancy related to COVID-19 vaccination is especially important and efforts are needed to understand the reasons for non-vaccination, in order to achieve a high coverage. Numerous previous studies, to date, have evaluated willingness and hesitancy about this vaccination, since it was first introduced among different groups [6,7,8,9,10,11]. In Italy, the intention to accept a COVID-19 vaccine with certainty in the general population ranged from 64.5% [12] to 84.1% [9], whereas in parents/guardians of children aged < 18 years old, the value was 60.4% [13], and in those aged 12–18 years, was 82.1% [14]. However, a relative paucity of data exists, at present, investigating parental willingness and hesitancy of COVID-19 vaccination for their children with chronic conditions [15,16].

Therefore, in this current epidemiological scenario, the present research is urgently needed to fill this gap in the existing literature. In accordance with this important scientific gap, the objectives of the present cross-sectional survey were to determine, among a sample of parents who had at least one child aged 5–11 years with a chronic medical condition in Italy, the willingness and hesitancy towards COVID-19 vaccination and the socio-demographic and COVID-19-related factors.

## 2. Materials and Methods

### 2.1. Setting and Sample

A telephone survey was conducted from 14 December 2021 to 4 January 2022, as part of larger COVID-19 vaccination research enrolling different populations [9,11,17,18]. Using a simple random sampling method, a total of 890 children between the ages of 5 and 11 years with a chronic condition were recruited from the medical records of the outpatient Department of Pediatrics of a Teaching Hospital which is located in the city of Naples, southern part of Italy. For each selected child, only one parent/guardian, aged 18 years old and over, was eligible to participate. No other inclusion criteria were applied.

The required sample size was calculated using a single proportion formula with the assumption that 50% of the subjects in the population were hesitant, which was used due to no study having been done in Italy, a margin of error of 5%, and a confidence interval of 95%. Finally, after accounting for a 10% non-response rate, the estimated total sample size became 427. This approach has been used in some of the previously mentioned studies on this topic [11,17].

### 2.2. Procedures

Ethical approval was granted from the Ethics Committee of the Teaching Hospital of the University of Campania “Luigi Vanvitelli”. During the study period, experienced interviewers made phone calls to the parents from Monday to Friday between 10:00 a.m. and 8:00 p.m. to avoid over-sampling non-working individuals. If the number called was not answered, two further attempts were made. Non-respondents were replaced with the next subsequent parent’s telephone number.

The interviewers, at the beginning of the dialogue with the parent who responded on the phone, stated that they were calling on behalf of the University of Campania, briefed the participants about the nature and scope of the research, the methodology, that their participation was on a voluntary basis, that all the collected information would be processed anonymously and confidentially, and that they could end their participation at any time without disclosing a reason. Verbal informed consent was obtained prior to progressing with the interview. No incentive for participation or survey completion was provided.

### 2.3. Data Collection

The questionnaire was developed based on previous surveys conducted by some of us on this topic among different populations [11,14,17,18]. Before the commencement of the data collection, a pilot study was performed on 10 individuals in order to test the participants’ understanding of the questionnaire items, the results of which were not taken into consideration for the study.

Parents were instructed to answer all specific questions in relation to their child aged 5 to 11 years with a chronic medical condition (hereafter referred to as ‘index child’). If respondent had more than one child with chronic medical condition within the target age, he/she was asked to refer to the child who was closest to 6 years of age (hereafter referred to as ‘index child’) or in the case of twins, he/she was requested to choose one and to refer to that child throughout the interview (hereafter referred to as ‘index child’).

The first section of the questionnaire assessed participants’ socio-demographic characteristics and other health-related information, including gender, age, marital status, level of education, occupation, partner’s occupation, number of children and of persons living in the same household, as well as whether they have had been infected by SARS-CoV-2, have cohabitants who were diagnosed with COVID-19, have been vaccinated against COVID-19 and whether the cohabitants in the household had already received the COVID-19 vaccine.

Regarding the index child, data were collected on gender, age, birth order, underlying chronic medical condition, and parents’ perception of the health status. The second section investigated the attitudes towards COVID-19 infection with two related statements, presented with a ten-point Likert scale for response, ranging from 1 = not at all concerned to 10 = concerned at all, regarding the perceived risk that the index child could be infected by SARS-CoV-2 and the belief that COVID-19 is a serious illness. The intention of participants to immunize the index child against COVID-19 was also assessed and open-ended questions were used to evaluate reasons for their willingness to vaccinate/not vaccinate, on a list of pre-designed responses. A respondent was allowed to give more than one reason for vaccinating/not vaccinating their child with chronic medical condition against COVID-19. To estimate the participants’ hesitancy regarding COVID-19, they were asked the five items of the PACV-5, a 5-item version of the validated 15-item Parent Attitudes About Childhood Vaccines Survey Tool, on a 5-point Likert scale [19,20]. A numeric score of 0–2 has been assigned to each item, with non-hesitant responses receiving a score of 0, responses of “not sure” and “I don’t know” receiving a score of 1, and hesitant responses receiving a score of 2. The total score ranged from 0 (non-hesitant) to 10 (most hesitant) per person and the level of hesitancy was categorized as low (0–4), moderate (5–6), and high (7–10). The third section queried participants’ sources of information related to the vaccine against COVID-19 for children with chronic conditions and whether they would like to receive additional information.

### 2.4. Statistical Analysis

First, descriptive statistics, including means and standard deviations for continuous variables and proportions for categorical variables, were used to describe the characteristics. Second, a series of bivariate analyses, the chi-square test and Student’s t-test, were used to determine the relationship between the different outcomes of interest and each of the explanatory continuous and categorical variables, respectively.

Third, independent variables with a *p*-value ≤ 0.25 in the bivariate analysis were introduced in the multivariate linear and logistic regression models to determine which variables maintained an independent association with the outcome. Four multivariate models were designed to determine the independent associations between predictors and the following outcome variables: parents’ belief that COVID-19 is a serious illness (Model 1); parents’ perceived risk that the index child could be infected with SARS-CoV-2 (Model 2); parents’ willingness to vaccinate their index child against COVID-19 (no = 0; yes = 1) (Model 3); COVID-19 vaccine parents’ hesitancy (PACV-5 score < 6 = 0; PACV-5 score 7–10 = 1) (Model 4).

The following independent variables regarding the respondent have been selected because they are potentially related to all outcomes: gender (male = 0; female = 1), age, in years (continuous), marital status (unmarried/separated/divorced/widowed = 0; married/cohabited with a partner = 1), baccalaureate/graduate degree (no = 0; yes = 1), employment (no = 0; yes = 1), partner’s employment (no = 0; yes = 1), number of children (1 = 0; >1 = 1), number of cohabitants (continuous), having been infected by SARS-CoV-2 (no = 0; yes = 1), have had at least one cohabitant who has been infected by SARS-CoV-2 (no = 0; yes = 1), having been vaccinated against COVID-19 (no = 0; yes = 1), at least one adult cohabitant who has been vaccinated against COVID-19 (no = 0; yes = 1), perception of the index child’s health status (continuous), having received information on vaccines against COVID-19 for children with chronic medical conditions from physicians (no = 0; yes = 1), and need of additional information on vaccines against COVID-19 for children with chronic medical conditions (no = 0; yes = 1). The following were the independent variables regarding the index child: age, in years (continuous), gender (male = 0; female = 1), severe chronic medical condition, such as diabetes, obesity, cancer and hemoglobinopathies (no = 0; yes = 1).

Values of *p* = 0.2 and *p* = 0.4 were used to select candidate variables for retention and exclusion in the final multivariate models. Odds ratios (ORs) and 95% confidence intervals (CIs) were used to estimate the strength of association in the logistic regression models, whereas standardized regression coefficients (β) in the linear regression models were calculated. For all analyses, two-tailed tests were used and a *p*-value equal to or less than 0.05 was considered statistically significant. The statistical analysis was performed with STATA, version 15.1 [21].

## 3. Results

### 3.1. Sample Characteristics

Of the total of 469 parents that were selected, 430 agreed to participate, resulting in a response rate of 91.7%. The principal characteristics of the survey participants and of the index child are summarized in Table 1. The distribution of the parents revealed the predominance of female (86.5%) and married/cohabited with a partner (90.7%) participants, the mean age was 40.5 years, more than two-thirds had completed higher education, more than half had two children, with the largest number being five. Respectively, 18.2% and 23.7% reported themselves and household members having tested positive for COVID-19, and only 12.1% said they had not been vaccinated. The mean age of the index child was 8.3 years, 52.8% were girls, 32.3% had diabetes, and the mean value of the health status rated by the parents was 8.5.

### 3.2. Attitude towards COVID-19

The parents’ belief that COVID-19 is a serious illness and the perceived risk that the index child could be infected with SARS-CoV-2, measured on a 10-point Likert scale, resulted, respectively, in a mean value of 8.8 and 8.5, with 56% in both cases who exhibited the higher score.

Table 2 presents the results from the multivariate linear and logistic regressions models, revealing the independent association of the different characteristics with the four outcomes of interest. Only two variables remained associated with the belief that COVID-19 is a serious illness. Respondents who did not have at least one cohabitant who had been infected by SARS-CoV-2 and had received the vaccination against COVID-19 had a higher concern (Model 1). The parents’ perceived risk that the index child could be infected by SARS-CoV-2 was higher in those who were more concerned about the severity of COVID-19. Respondents with a younger index child, who did not have at least one cohabitant who had tested positive for COVID-19, were less worried (Model 2).

A total of 38.8% of participants expressed their willingness to have their child vaccinated against COVID-19, whereas 37% were unsure and 24.2% reported that their child would not receive the vaccine. Among the parents who reported a positive intention, the main motivating reasons given in support of this intention included protecting their child’s health (81.5%), followed by the perception that the child is at risk (42.2%), and that COVID-19 is a severe disease (38.7%). Common reasons reported by respondents that did not intend, or were uncertain, to vaccinate their child included the concern about possible side effects (65.4% and 45.9%) and scarce knowledge about COVID-19 vaccination in children (43.3% and 57.1%).

The multivariate logistic regression model showed that seven variables were significantly and independently associated with the increased likelihood of exhibiting willingness to have their child vaccinated against COVID-19. Parents who did not need additional information regarding the COVID-19 vaccination for children with chronic medical conditions (OR = 0.38; 95% CI = 0.24–0.62), those with a higher level of education (OR = 2.23; 95% CI = 1.32–3.77), those who have been vaccinated against COVID-19 (OR = 4.76; 95% CI = 1.76–12.85), those whose index child was older (OR = 1.18; 95% CI = 1.05–1.32), those who had received information on this vaccination from physicians (OR = 1.95; 95% CI = 1.21–3.17), those with a higher concern about the severity of COVID-19 (OR = 1.27; 95% CI = 1.07–1.52), and those who had a higher perceived risk that their child could be infected by SARS-CoV-2 (OR = 1.19; 95% CI = 1.04–1.36), were more likely to express a greater willingness to vaccinate their child (Model 3 in Table 2).

### 3.3. COVID-19 Vaccine Hesitancy

Overall, the mean PACV-5 score among participants was 4.8, with 26.3% identified as high-hesitant towards anti-COVID-19 vaccination, scoring ≥ 7, 27.9% as moderate-hesitant, scoring 5–6, and 45.8% as low-hesitant, scoring ≤ 4.

The frequencies of parents with a PACV-5 score of 0 and 10 were 6.3% and 4.2, respectively. Responses to the individual items of the PACV-5 are shown in Table 3. More than one-third (39.1%) of the parents agreed or were not sure that a child should get more vaccines that are good for them, almost two-thirds (61.5%) thought that fewer vaccines at the same time were better for children, and only 60.9% strongly disagreed or disagreed that it is better for children to develop immunity by getting sick than to get a shot. Less than one-third (24.9%) of respondents considered themselves to be vaccine hesitant. Less than half (44.5%) trusted the information provided to them regarding the COVID-19 vaccine.

Respondents with a lower level of education (OR = 0.32; 95% CI = 0.15–0.72), those who were less concerned about the severity of the disease (OR = 0.75; 95% CI = 0.64–0.88), those who did not get the COVID-19 vaccine (OR = 0.24; 95% CI = 0.12–0.47), and those who were less worried that the index child could be infected by SARS-CoV-2 (OR = 0.87; 95% CI = 0.77–0.99), were more likely to be high-hesitant (Model 4 in Table 2).

### 3.4. Information Sources

In the final questions, the researchers enquired about participants’ sources of information, regarding vaccines against COVID-19 for children with chronic conditions, and almost all respondents (98.9%) reported that they had gained this information.

The most trusted source of information was physicians (65.8%), followed by mass media (47.2%), and Internet (38.6%). More than two-thirds of the sample (69.8%) wanted to know more about the vaccine against COVID-19 for children with chronic conditions.

## 4. Discussion

To the best of our knowledge, this is the first survey on a population of parents of children aged 5–11 years with chronic conditions in Italy, which described the willingness and the hesitancy regarding COVID-19 vaccination and the influencing factors. The knowledge about willingness and hesitancy, and the associated predictors, plays a crucial role in COVID-19 prevention and management.

It is important to highlight findings from the present study, where only a little more than one-third (38.8%) of the participating parents said that they would vaccinate their children against COVID-19, and it is also evident that the hesitancy is widely prevalent, with more than one-quarter (26.3%) found to be high-hesitant, with a PACV-5 score ≥ 7, having an impact on the COVID-19 vaccine uptake among the targeted population.

The value of the willingness is unsatisfactory, and it is below a recent estimate of 49.9% in China, among parents of children with special diseases, with a mean age of 1.4 years, and more willingness was found for those with congenital heart diseases [16] and it is higher of 23% observed in Taiwan among caregivers of children with a mean age of 11.3 years with attention-deficit/hyperactivity disorder [15]. Other surveys among parents of healthy children of similar age have observed considerably higher prevalence. Indeed, the willingness towards the vaccination was 93.6% for children aged 5–8 years in Brazil [22], 87.9% among 9–11-year-olds in Canada [23], 86.75% for 3–6 years in China [24], 65.2% for children with a median age of 7.5 years in a multicenter survey in the United States, Canada, Israel, Spain, and Switzerland [25], 45.9% for those aged 5–10 years in the United States [26], and 42.9% for children with an average age of 7.4 years in Japan [27]. Moreover, prevalence of the participants that were found to be vaccine high-hesitant was higher than the value of 15.7%, reported in a study that used the PACV-15 tool in China [28], and 12.4% with the PACV-5 in Italy [14]. It has been observed that the issue parents were most concerned about was that, for almost two-thirds, it is better for children to get fewer vaccines at the same time, and one-third considered themselves to be COVID-19 vaccine high-hesitant. Hence, it is imperative to first identify these vaccine hesitant groups and their doubts and concerns, and strategically work towards restoring their trust and belief towards this vaccination, in order to ensure better immunization coverage in the future. However, it is necessary to be cautious in comparing the results with those of recent similar published studies due to the differences, for example, in the period of the study conducted, sampling methodologies, population socio-demographic characteristics, and data collection instruments and procedures.

The influence of the different sources of information regarding vaccines against COVID-19 for children with chronic conditions on the willingness and hesitancy towards COVID-19 vaccination was also investigated, since the information can play a key role in determining an individual’s health actions. It is very important to note that among the respondents, the physicians were viewed as the most common source of reliable health information. Physicians are the ideal health care worker to impart vaccination-related information to the parents and they have an influential role regarding advocacy of vaccines. In the present study, parents who reported the physicians as a source of information were more willing to vaccinate their child than parents who heard about this vaccine through other sources. Nowadays, a sizable body of literature, which found that receiving information and recommendation on vaccination from physicians is one of the most significant and consistent factors that influences the audience’s decision, supports this finding. Indeed, it has been revealed that trust in physicians, as in government organizations and scientific journals, positively influences the level of knowledge, the vaccine confidence, and the vaccine uptake [18,29,30,31,32].

Another interesting finding in this study was that parents were more likely to report that they would vaccinate their child if they did not need additional information about this vaccination, compared to parents with this need. Parents who were unwilling or uncertain to vaccinate their child should understand the risks and need to know how to make it less likely that their child gets an infection, as well as the greater risk that infection will spread in the community. Information generated from this study underlined the need to identify policies and public health educational programs that could be implemented to reduce hesitancy and increase vaccination uptake. Moreover, these observed results also underlined the fact that physicians should discuss vaccination with the family and provide them effective communication, in order to explain understandable and evidence-based information on the benefits and risks of this vaccination, acknowledging parental concerns and correcting the misconceptions. Mass media and Internet were the two main additional sources to obtain information, and this is of partial concern, because many people make decisions based on their information, although inaccurate, misleading, and unconfirmed information by the respective specialists can be circulated online [33,34,35]. These sources cannot replace physicians, as all other scientific sources, since they should be the first point of contact for parents regarding the importance of this vaccination for their children.

It is of interest to note that in this study, the main reasons cited why the parents were willing to vaccinate their children were to provide protection from COVID-19 to the person being vaccinated and the perceived risk that the child is at risk. It is worth mentioning that less than one-third of the respondents indicated the trust in the efficacy of the vaccine. Moreover, in the present study, participants raised a number of reasons to explain their refusal or indecision to have their children vaccinated. These included lack of knowledge about the vaccine and fear of eventual adverse effects. Consequently, the presence of such barriers indicates a general adverse attitude towards COVID-19 vaccines and this finding should help pediatricians focus on issues of concern to parents that can be stressed in a child’s routine preventive health care. Therefore, identifying and alleviating these obstacles is the second most important step in parents’ education in COVID-19 vaccination. Barriers among parents who were unwilling or uncertain to vaccinate their children may be harder to address and concerns about side effects constituted a critical theme, which is a commonly cited key reason, globally, for vaccine hesitancy and refusal [14,28,36,37,38].

It should be underlined that in both Western and Asian countries, it has been widely reported that the positive emotion of the public towards the safety of the COVID-19 vaccine was associated with increased vaccination uptake [39,40,41,42]. This highlights the need for comprehensive and coordinated health promotion campaigns, to improve understanding of the importance of vaccines and efforts of healthcare providers about the COVID-19 vaccine among this population, to equip them with easily understandable information. It should be underlined that the vaccination has an excellent safety profile and that there is no evidence of serious vaccine-related adverse events, and this will enable the public to make informed choices regarding this vaccination.

There are some additional interesting findings about the effects of several variables on the different outcomes of interest, explored through multivariate linear and logistic regression analysis in the current study. First, among the several socio-demographic characteristics of the sample, only education remained as associated factor for parents’ decisions to vaccinate their children against COVID-19, since their college degree or higher level of education was significantly associated with the willingness to have their child vaccinated against COVID-19, and lower education was associated with a high level of hesitancy. This is consistent with data showing that an educational level below a bachelor’s degree predicted willingness and hesitancy towards COVID-19 vaccination [8,14,43,44,45]. Second, respondents who were not aware of someone in the household that had been infected by SARS-CoV-2 were less likely to be concerned about the severity of the disease and more likely to report a lower risk perception that their child could be infected, when compared with those who did know of anyone that had been infected. These findings are not surprising and were similar to other studies with participants who knew someone with severe COVID-19 symptoms or had died, resulting in more willingness to get vaccinated than others [46,47]. However, it is not expedient to wait for someone to get infected with COVID-19 or to die before getting vaccinated for COVID-19. Third, willingness to vaccinate was found to be associated with the perceived risk for their children of being infected with SARS-CoV-2, and high-hesitancy, with a lower perceived risk. These associations were in the same direction to that hypothesized before the study, since respondents who define themselves or their children at a higher risk, although perceived, should be more worried with an increased perceived risk of exposure to COVID-19 and, therefore, they should be more willing or less hesitant [24,48,49,50]. Fourth, parents’ concern about the severity of the disease was associated with the willingness and the hesitancy toward this vaccination, with those with a higher concern being more likely to be willing and less hesitant. This finding is supported by other evidence reported in the literature [51,52,53]. Lastly, vaccinated parents reported that they were more willing to have their children vaccinated than those who were unvaccinated. This finding seems to be consistent with previous studies, which observed that the parents’ vaccination or intention to vaccinate themselves against COVID-19 had a significant positive impact on the willingness of the COVID-19 vaccine for the children [26,54,55,56].

The present survey had potential methodological limitations that should be mentioned and discussed in interpreting its findings. First, this survey used a cross-sectional study design, which limits the possibility of making inferences on the temporality and causality of the observed relationships between variables. Second, this was a single-institution study and, thus, it is possible that the results that have been obtained may not be generalizable to the nationwide situation. Third, data were collected with a self-reported questionnaire format, so might be subject to recall or social-desirability bias, since participants might have consciously selected responses that were positively oriented towards vaccination and appropriate behaviors, thus, overestimating the adherence to COVID-19 preventive measures. However, the questionnaire did not include any identifiable data, and participants were assured that all identities would be kept anonymous. As such, the findings are likely to be authentic. The limitations notwithstanding, this survey provides an important contribution to this topic and the information will be useful for planning and implementing health education strategies.

## 5. Conclusions

In summary, the results of this current cross-sectional survey provide a foundational understanding of parents’ willingness and hesitancy, regarding COVID-19 vaccination for their children with chronic conditions, in the context of a large infectious disease pandemic. The willingness to get this vaccination appeared unacceptable. The major reasons behind COVID-19 vaccine refusal were related to a lack of knowledge and fear of eventual adverse effects. These findings could be used to target public health interventions and a concerted effort is needed to educate parents and to enhance their awareness, which could improve their acceptance of the vaccination and, consequently, protect this high-risk group of children and reduce the spread of SARS-CoV-2.

## Figures and Tables

**Table 1:** Socio-demographic and key characteristics of the study population.

	N	%
*Parent*		
Age, years	40.5 ± 6.1 (25–57) *	
Gender		
Female	372	86.5
Male	58	13.5
Marital status		
Unmarried/separated/divorced/widowed	40	9.3
Married/cohabited with a partner	390	90.7
Number of children		
1	83	19.3
>1	347	80.7
Educational level		
High school degree or less	337	78.4
Baccalaureate/graduate degree	93	21.6
Employment status		
Unemployed	218	50.7
Employed	212	49.3
Partner’s employment status		
Unemployed	91	21.2
Employed	339	78.8
Having cohabitants		
1–3	269	62.6
>3	161	37.4
Having been infected by SARS-CoV-2		
No	352	81.8
Yes	78	18.2
Having at least one cohabitant who had been infected by SARS-CoV-2		
No	328	76.3
Yes	102	23.7
Vaccinated against COVID-19		
No	52	12.1
Yes	378	87.9
At least an adult cohabitant vaccinated against COVID-19		
No	57	13.3
Yes	373	86.7
*Index child*		
Age, years	8.3 ± 1.9 (5–11) *	
Gender		
Female	227	52.8
Male	203	47.2
Birth order		
First	226	52.6
Second	140	32.6
≥Third	64	14.8
Underlying chronic medical conditions		
Diabetes	139	32.3
Endocrine	114	26.5
Kidney	46	10.7
Respiratory	29	6.8
Rheumatic	27	6.3
Gastrointestinal	22	5.1
Nutrition-related	19	4.4
Hematologic	18	4.2
Oncologic	16	3.7
Parents’ rate health status	8.5 ± 1.4 (1–10) *	

* Mean ± Standard deviation (range).

**Table 2:** Multivariate linear and logistic regression analysis results examining the outcomes of interest according to several explanatory variables.

Variable	Coeff.	SE	*t*	*p*
Model 1. Parents’ self-reported concern about the severity of COVID-19 F (5, 424) = 4.45, *p* = 0.0006, R^2^ = 5%, adjusted R^2^ = 3.9%				
Not having at least one cohabitant who have been infected by SARS-CoV-2	−0.45	0.18	−2.48	0.014
Having been vaccinated against COVID-19	0.49	0.24	2.03	0.043
Children with severe underlying chronic medical conditions	0.28	0.16	1.81	0.072
Having received information on vaccine against COVID-19 for children with chronic medical conditions from physicians	0.29	0.17	1.76	0.079
Need of additional information on vaccine against COVID-19 for children with chronic medical conditions	0.22	0.17	1.33	0.186
Model 2. Parent’s perceived risk that the index child could be infected by SARS-CoV-2 F (9, 420) = 20.03, *p* < 0.0001, R^2^ = 30%, adjusted R^2^ = 28.5%				
Concerned about the severity of COVID-19	0.61	0.05	11.26	<0.001
Younger index children	−0.12	0.04	−2.61	0.009
Not having at least one cohabitant who had been infected by SARS-CoV-2	−0.45	0.20	−2.20	0.029
Having at least one adult cohabitant vaccinated against COVID-19	0.49	0.28	1.76	0.079
Unemployed	−0.32	0.20	−1.58	0.115
Having been vaccinated against COVID-19	0.46	0.30	1.54	0.125
High school degree or less	−0.35	0.23	−1.50	0.134
Female	0.39	0.27	1.44	0.151
Need of additional information on vaccine against COVID-19 for children with chronic medical conditions	0.22	0.19	1.18	0.24
	OR	SE	95% CI	*p*
Model 3. Parents’ willingness to vaccinate the index child against COVID-19 Log likelihood = −245.79, χ^2^ = 82.91 (7 df), *p* < 0.0001				
No need of additional information on vaccine against COVID-19 for children with chronic medical conditions	0.38	0.09	0.24–0.62	<0.001
Having been vaccinated against COVID-19	4.76	2.41	1.76–12.85	0.002
Baccalaureate/graduate degree	2.23	0.59	1.32–3.77	0.003
Older index children	1.18	0.07	1.05–1.32	0.004
Having received information on vaccine against COVID-19 for children with chronic medical conditions from physicians	1.95	0.48	1.21–3.17	0.006
Higher self-reported concern about the severity of COVID-19	1.27	0.11	1.07–1.52	0.006
Higher perceived risk that the index child could be infected by SARS-CoV-2	1.19	0.08	1.04–1.36	0.009
Model 4. Parent’s hesitancy regarding COVID-19 vaccination for the index child Log likelihood = −205.45, χ^2^ = 84.42 (7 df), *p* < 0.0001				
Not having been vaccinated against COVID-19	0.24	0.08	0.12–0.47	<0.001
Lower self-reported concern about the severity of COVID-19	0.75	0.06	0.64–0.88	0.001
High school degree or less	0.32	0.13	0.15–0.72	0.006
Lower perceived risk that the index child could be infected by SARS-CoV-2	0.87	0.05	0.77–0.99	0.036
Having been infected by SARS-CoV-2	1.74	0.52	0.96–3.14	0.066
Unemployed	0.68	0.18	0.40–1.17	0.17
Younger index children	0.97	0.02	0.94–1.01	0.292

**Table 3:** Distribution of COVID-19 vaccine hesitancy using the PACV-5 survey tool.

PACV-5 Survey Items	N (%)
*Children get more shots than are good for them*	
Strongly agree/Agree	119 (27.7)
Not sure	49 (11.4)
Strongly disagree/Disagree	262 (60.9)
*It is better for my child to develop immunity by getting sick than to get a shot*	
Strongly agree/Agree	73 (17)
Not sure	95 (22.1)
Strongly disagree/Disagree	262 (60.9)
*It is better for children to get fewer shots at the same time*	
Strongly agree/Agree	264 (61.5)
Not sure	81 (18.8)
Strongly disagree/Disagree	85 (19.7)
*Overall, how hesitant about the COVID-19 vaccination for your child would you consider yourself to be?*	
Very hesitant/Somewhat hesitant	107 (24.9)
Not sure	49 (11.4)
Not hesitant at all/Not too hesitant	274 (63.7)
*I trust the information I receive about the COVID-19 vaccination*	
Strongly agree/Agree	191 (44.5)
Not sure	142 (33)
Strongly disagree/Disagree	97 (22.5)

## Data Availability

The data presented in this study are available on request from the corresponding author.
